# Disrupting VEGF–VEGFR1 Interaction: De Novo Designed Linear Helical Peptides to Mimic the VEGF_13-25_ Fragment

**DOI:** 10.3390/molecules22111846

**Published:** 2017-10-28

**Authors:** Beatriz Balsera, M. Ángeles Bonache, Marie Reille-Seroussi, Nathalie Gagey-Eilstein, Michel Vidal, Rosario González-Muñiz, María Jesús Pérez de Vega

**Affiliations:** 1 Instituto de Química-Médica (IQM-CSIC), Juan de la Cierva 3, Madrid 28006, Spain; bea.balsera@gmail.com (B.B.); angelesbonache@iqm.csic.es (M.Á.B.); iqmg313@iqm.csic.es (R.G.-M.); 2 UMR 8638 CNRS, UFR de Pharmacie, Université Paris Descartes, PRES Sorbonne Paris Cité, 4 avenue de l’Observatoire, Paris 75006, France; marie.reille@gmail.com (M.R.-S.); nathalie.eilstein@parisdescartes.fr (N.G.-E.); michel.vidal@parisdescartes.fr (M.V.); 3 UF “Pharmacocinétique et Pharmacochimie”, Hôpital Cochin, AP-HP, 27 rue du Faubourg Saint Jacques, Paris 75014, France

**Keywords:** peptides, α-helix, protein–protein interactions, VEGF-VEGFR1, angiogenesis

## Abstract

The interaction between vascular endothelial growth factor (VEGF) and its receptors (VEGFR) has important implications in angiogenesis and cancer, which moved us to search for peptide derivatives able to block this protein–protein interaction. In a previous work we had described a collection of linear 13-mer peptides specially designed to adopt helical conformations (Ac-SSEE**X^5^**ARN**X^9^**AA**X^12^**N-NH_2_), as well as the evaluation of seven library components for the inhibition of the interaction of VEGF with its Receptor 1 (VEGFR1). This study led to the discovery of some new, quite potent inhibitors of this protein–protein system. The results we found prompted us to extend the study to other peptides of the library. We describe here the evaluation of a new selection of peptides from the initial library that allow us to identify new VEGF-VEGFR1 inhibitors. Among them, the peptide sequence containing F, W, and I residues at the 5, 9, and 12 positions, show a very significant nanomolar IC_50_ value, competing with VEGF for its receptor 1, VEGFR1 (Flt-1), which could represent a new tool within the therapeutic arsenal for cancer detection and therapy.

## 1. Introduction

Protein–protein interactions (PPIs) play essential roles in multiple biological functions mediating both in physiological and pathological processes and constitute important targets in biological and medicinal chemistry. Aberrant or inappropriate interactions may be associated with the pathogenesis of several diseases; therefore, the modulation of certain PPIs constitutes a challenging approach for therapeutic intervention in pathological situations [1]. In recent years, a lot of work in this direction has been reported describing different PPI disrupting agents both small molecules and peptides [2,3,4,5]. Some of them have even progressed to clinical development, especially for the inhibition of the interaction of p53 with its suppressor HDM2 (Human Double Minute 2) which is highly implicated in cancer [6,7,8]. A validated approach to tackle the problem of disrupting PPIs is to use peptides to mimic the protein surfaces involved in the interaction, with the aim of reproducing the secondary structure of the hot-spot, an interface region contributing most to the PPI [9,10]. Recent technical advances concerning peptide synthesis and delivery have permitted a resurgence of peptide drug discovery and development [11,12,13,14]. Peptides also show some advantages, like their chemical flexibility, which permits them to adapt to the large and shallow protein surfaces that are generally involved in their interaction with other proteins. Peptides are not only able to mimic the structural features of the protein interfaces, they are also more biocompatible, which redounds in lower toxicity. 

We had previously described a collection of linear 13-mer peptide library, de novo designed for adopting helical conformations. Our hypothesis was based on the fact that α-helices are the secondary structure element most frequently involved in PPIs [15]. They were designed to fix a combination of either three aromatic—or two aromatic and one aliphatic—residues on one face of the helix (Ac-SSEE**X^5^**ARN**X^9^**AA**X^12^**N-NH_2_), taking into account that frequently only the hydrophobic face of the helix is involved in binding, and also that key residues for affinity are located at relative positions *i*, *i* + 3(*i* + 4), *i* + 7. The library was conceived as a tool to identify peptides able to disrupt PPIs in which helical motives were involved, playing an essential role. In order to validate our initial hypothesis some peptides were selected to evaluate their capacity to interfere with two well-studied PPI targets, such as the p53-MDM2 and VEGF-VEGFR1 interactions. In both cases, the protein–protein contact takes place through interfaces in which the mediation of a hydrophobic α-helix is essential. The results of the appropriate binding experiments showed that some of the tested peptides were able to mimic p53 and VEGF ‘hot-spots’, binding with their complementary proteins MDM2 (murine form of the HDM2 suppressor factor) and VEGFR1, respectively, and finally hampering their interaction with the respective partners, p53 and VEGF [15].

The interaction of the vascular endothelial growth factor (VEGF) with its receptors, is a triggering factor of angiogenesis, a physiological process of generation of blood vessels. Angiogenesis plays also an important role in pathological situations being closely related to certain diseases like cancer and metastasis [16]. VEGF is one of the best known proangiogenic factors, and its biological action is mediated by its binding to specific receptors that are transmembrane proteins with tyrosine kinase activity, VEGFR1, VEGFR2, and VEGFR3 [17,18]. One of the three main epitopes identified as essential for the VEGF-VEGFR1 interaction is the fragment VEGF_17-25_, located at the N-terminal of VEGF. Within this fragment, Phe^17^ appears to be a key residue for the VEGF–VEGFR1 interaction together with Tyr^21^ and Tyr^25^ residues, which are important for the stabilization of the helical secondary structure [19]. Related to this, we had previously described 13-mer linear and cyclic peptides derived from this fragment that were able to bind to VEGFR1, showing IC_50_ values in the micromolar range [20].

To validate our helical library in the VEGF/VEGFR system [15], seven peptides of the whole collection were initially selected, including the FYY containing derivative as the most related to VEGF_17-25_ fragment. Six of them had in their sequence three aromatic residues located at relative positions *i*, *i* + 4, *i* + 7, corresponding to positions 5, 9, 12 of the sequence (Ac-SSEE**X^5^**ARN**X^9^**AA**X^12^**N-NH_2_) [15]. It is worth to notice that the relative position of the key residues in VEGF_17-25_ fragment, namely F*^i^*Y^(*i*+4)^Y^(*i*+8)^, is not exactly the same that in the library components, but the fact that the three aromatic residues are located at the same face of the helix could permit that they mimic the native helical fragment. When these compounds were tested for their ability to displace biotinylated VEGF_165_ bound to the extracellular domain of VEGFR1, at the unique dose of 100 μM, (compounds **1**–**7**, Table 1), inhibition values in the range of 30–50% were found for most of them [15]. Two compounds, **2** (FYW) and **3** (FWY), showed good IC_50_ values (29 ± 3 μM and 23 ± 4 μM, respectively), comparable to that of model peptide QK (Table 1). These results already suggested that the single substitution of Tyr^9^ and Tyr^12^ residues by Trp, has some advantages leading to better inhibitors of the VEGF-VEGFR1 interaction. On the contrary, peptide **7** with the combination FYI, resulting from the substitution of Y^12^ by an aliphatic Ile residue, was inactive. 

These results were promising enough to prompt us to extend the study to other peptides from our library [15]. Here we describe the evaluation of the whole sub-library of peptides having three aromatic residues at the mentioned positions, and some components with two aromatic and one aliphatic residues. This study led to the identification of a peptide sequence, containing F, W, and I residues at the 5, 9, and 12 positions, showing submicromolar IC_50_ value, competing with VEGF for its receptor 1, VEGFR1.

## 2. Results and Discussion

The binding studies were performed on VEGF isoform VEGF-A (VEGF165) that is the most commonly involved in pathological angiogenesis. Compounds were first evaluated for their ability to displace biotinylated VEGF-A at two different doses, 30 and 100 μM (Table 2). A chemiluminescent assay, relying on competition between tested compounds and biotinylated VEGF-A for binding to the extracellular domain of recombinant VEGFR1, was used [22]. At the lower dose of 30 μM, more than half of the compounds showed inhibition percentage values higher that 40%. 

Considering the displacement percentage values at the dose of 100 μM of compound tested, in general it can be said that the introduction of a Trp residue at the key positions enhances the capacity of the peptide to bind to the VEGFR1 receptor. Thus, considering the previously described peptides (Table 1), compounds **2** (FYW), **3** (FWY), and **4** (WYY) resulting from the substitution by Trp of residues at key positions 12, 9, and 5 respectively, display about 15–20% higher binding affinity than peptide **1**, keeping the natural sequence FYY (29 ± 3%). Similar results are also observed when comparing compound **9** (FFF, 37 ± 3%), with peptides **10** (WFF, 55 ± 1%), **12** (FWF, 55 ± 1), and **22** (FFW, 82 ± 1), all showing higher inhibitory values, especially in the case of **22** (Table 2). The same tendency is observed in the case of **15** (FYF, 27 ± 5%), and its analogues **16** (WYF, 62 ± 1%), **12** (FWF, 55 ± 1%), and **2** (FYW, 48 ± 6%) (Table 1 and Table 2). In contrast, in the case of **32** (FIY, 60 ± 1%) and **30** (FIW, 47 ± 6%) a decrease in the activity is observed due to the replacement of Tyr^12^ by Trp. Regarding the substitution by aliphatic residues, it seems that the introduction of Ile at key position 9 is better tolerated than at position 12, as it can be inferred by looking at the inhibitory values of compounds **1** (FYY, 29 ± 3%), **32** (FIY, 60 ± 1%), and **7** (FYI, 0%). Less tolerated was the incorporation of Leu residues at the same positions, leading to poorly active or inactive peptides, **33** (FLY, 19 ± 7%) and **31** (FYL, n.a.). The simultaneous introduction of two Trp residues in the same sequence—like in **20** (FWW, 43 ± 3%), **23** (WYW, 50 ± 1%), and **27** (WWY, 64 ± 5%)—also redounds in better binding results, compared to peptide **1** (FYY, 29 ± 3%), but in general these compounds do not improve the affinity of the mono-Trp-substituted peptides. Although, one of the best results is shown by compound **21**, with three Trp residues at the key positions (WWW, 78 ± 2), this result was not corroborated afterwards. Compounds **1**, **4**, and **6**, differing in the aromatic residue at position 5 are almost equipotent. Compared to peptide **9** (FFF, 37 ± 3), equipotent to model **1**, the incorporation of a single Tyr residue is detrimental at position 5, compound **11** (7 ± 4), and conservative at positions 9 and 12, compounds **15** (27 ± 5) and **5** (39 ± 6), respectively, as it occurs also for the two-Tyr-containing analogues **17** (6 ± 1) and **26** (38 ± 4) (Table 1 and Table 2). Most of the above commented results are corroborated when the compounds were tested at a 30 μM concentration.

To summarize, from the above SAR, it seems that, in general, aromatic amino acids are suitable at position 5, with some exceptions (compounds **11** and **17**, both having Phe at position 12). Concerning position 9, the replacement of Tyr^9^ by Ile, Trp or Phe is well tolerated, as it can be seen for compounds **32** (FIY, 60 ± 1%), **3** (FWY, 50 ± 5%), and **5** (FFY 39 ± 6%) respectively, while Leu seems to be detrimental for activity, **33** (FLY, 19 ± 7%). Regarding position 12, replacement by Phe apparently has no consequences, **15** (FYF, 27 ± 5%), while introduction of Trp gives a better result, **2** (FYW, 48 ± 5%), and Ile leads to an inactive compound **7** (FYI, n.a.). An exception to this is the high inhibition value found for peptide **29** (FWI, 72 ± 1), leading to a remarkable result despite that an Ile residue is in the place of Tyr 12. In this case, position 9 is occupied by a Trp that, as already commented, seems to increase the affinity for the receptor. On the whole, with the data in our hands it can be tentatively said that the incorporation of Trp at any of the positions give higher inhibition percentages.

Our results concerning the replacement of some aromatic residues by Trp correlate with the work of D´Andrea and co-workers that reported two peptides having the W_17_Y_21_Y_25_ motive and able to bind with high affinity to VEGFR1. In these cases, Phe17 had been replaced by Trp, resulting in very active peptides. However, one of them is a pro-angiogenic agent instead of showing the expected antiangiogenic activity as inhibitor of the VEGF-VEGFR1 interaction (peptide QK, Table 1) [21,23], while the other behaves as an anti-angiogenic [24]. The results are also in certain agreement with the data of a parallel study recently published by some of us, that describes short peptide analogs of the native sequences of the N-terminal α-helix of VEGF-A, VEGF-B, and PLGF, all of them natural ligands of VEGFR1. Sequence alignment showed that key residues for binding to VEGFR1 are respectively: F_17_Y_21_Y_25_, W_17_Y_21_, and F_25_W_29_Y_33_. These results also pointed to the benefit that the substitution by the Trp amino acid can have for binding [25].

To avoid false positive results due to possible contamination with metals or peptide aggregation [26,27], the screening was repeated at the same dose of 30 μM, but in the presence of EDTA, for those compounds having inhibition values above 60% (Table 2). The new experiments performed in the presence of EDTA led to slightly lower inhibition percentage values, especially for peptides with high Trp content. These last more reliable values were used to select the best peptides for dose–response experiments and IC_50_ determination. Only seven compounds exhibiting percentages of inhibition larger than 40% were studied (Table 3). 

**Figure 1:**
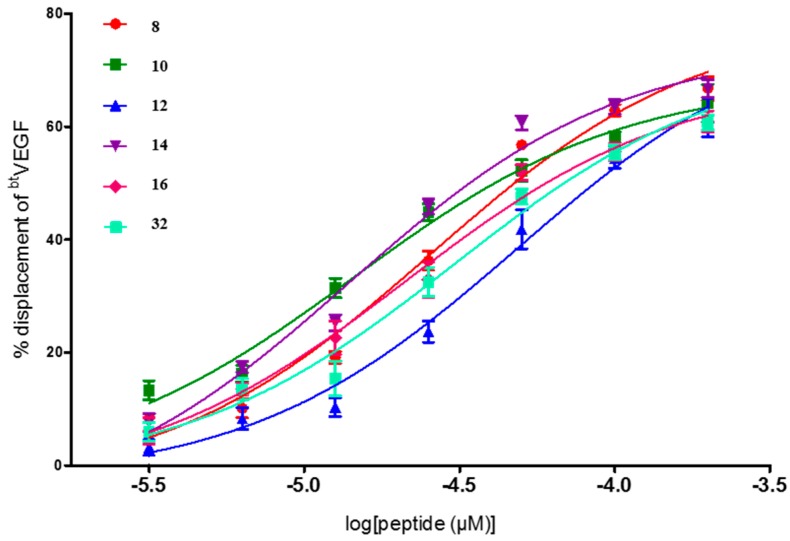
Dose–response curves for 8, 10, 12, 14, 16, and 32 peptides on VEGFR-1 displacement assays. Curves were fitted with log(inhibitor) vs. response method using GraphPad Prism.

**Figure 2:**
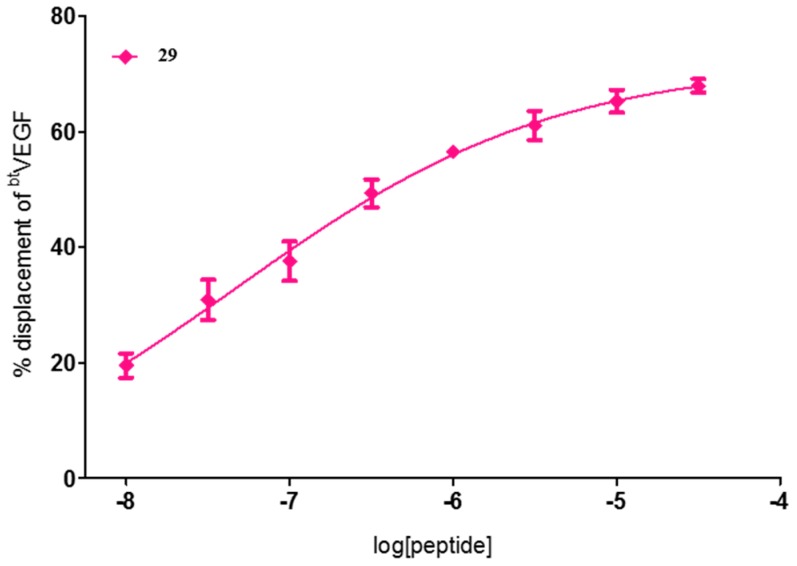
Dose–response curve for compound **29**.

From the seven compounds studied to find the IC_50_ values, five of them had only one Trp residue in the sequence occupying either the 5 or the 9 position, (**10**, **12**, **14**, **16**, and **29**), one has two Trp residues (**8**), and two have one aliphatic residue of Ile, **29** and **32**, at positions 12 and 9, respectively. All of them showed good IC_50_ values, being peptides **10**, **14**, and **29** the most effective as blockers of the VEGF-VEGFR1 interaction within this series (Figure 1 and Figure 2). All three have a Trp residue, either at position 5 or 9. Quite outstanding is the result found for compound **29**, FWI, which showed submicromolar activity with an excellent IC_50_ value of 0.05 μM (Figure 2). These results correlate with the above commented finding about the replacement of only one of the key amino acid residues by Trp, which seems to be beneficial for increasing the affinity for VEGFR1. Compound **29**, in addition to the Trp^9^, has an aliphatic Ile at position 12, in replacement of the native residue of Tyr^12^ (that would corresponds to Tyr^25^ of the native VEGF sequence). Tyr25 residue was suggested to play a relevant role for stabilization of the helix, more than being essential for the interaction with VEGF receptors [19]. The IC_50_ value found for **29** is as far as we know the best VEGFR1 affinity value ever reported for a peptide, as it can be seen by the results found for the above mentioned related peptides. Just to compare, it can be cited that the proangiogenic peptide QK described by D’Andrea and co-workers, Ac-KLTWMELYQLAYKGI-NH_2_ [21], in our assay shows an IC_50_ = 32 ± 8 μM [15]. In addition, the best result obtained for a small collection of peptides recently described by Wang, L. et al., corresponding to the cyclic peptide Ac-[CTVELMGTVAKQLVPC]-NH_2_, displayed an IC_50_ value of 10.4 ± 2.8 μM [25].

In summary, the affinities found suggest that the presence of a Trp residue, especially at positions 5 and 9, which is an important feature for VEGFR1 recognition, leading to linear peptides able to bind to this receptor with high affinity. The ultimate result of this study is the discovery of a linear 13-mer peptide **29**, that shows the highest binding activity hitherto reported for a VEGFR1 peptide ligand (IC_50_ = 0.05 μM). To confirm this outstanding result we planned to perform a more complete pharmacological characterization of peptide **29** to explore its potential application in cancer detection and therapy. 

## 3. Experimental Section

### 3.1. Synthesis

The library peptides were conveniently synthesized by parallel solid-phase methodologies and are described in [15].

Briefly, peptides were prepared starting from a Rink amide resin (0.34 g/mol) following the Fmoc/^t^Bu strategy. Most of them were prepared manually, repeating the same procedure to introduce each amino acid. Swelling of the resin was performed with DMF and DCM (1 mL/100 mg of resin, 30 s × 4). Fmoc deprotections were performed with 20% piperidine in DMF (1 mL/100 mg of resin, one wash for 1 min and three for 10 min). Coupling reaction was carried out with HCTU (2 equiv), DIEA (2 equiv) and the corresponding Fmoc amino acid (2 equiv) in DMF for 1 h to obtain the peptides. Each coupling was checked by the Kaiser test and repeated if necessary. Some of the peptides were prepared using an automatic synthesizer coupled to a microwave heater (Cem Liberty1). In this case, resin was swelled with DCM for 10 min. Fmoc deprotections were performed with 20% piperidine in DMF in two steps. The first step was performed at 40 °C for 30 s and the second at 75 °C for 5 min. Coupling reactions were performed at 75 °C using Fmoc amino acids in DMF (5 equiv related to the resin), HBTU/HOBT in DMF (5 equiv), and DIEA in NMP (10 equiv).

Acetylation reactions were performed with DIEA (20 equiv) and Ac_2_O (20 equiv) in DMF for 1 h or using an Ac_2_O/DIEA/DMF (1:1:1) solution (4 × 10 min).

Cleavage of peptides from the resin, and concomitant side chain deprotection, were performed using TFA/EDT/H_2_O/TIPS (94:2.5:2.5:1) (1 mL, 100 mg of resin) at room temperature for 3 h. The resin was filtered off and crude products were precipitated with cold Et_2_O. The resulting solid was centrifuged, washed twice with ethyl ether, and then lyophilized. 

Peptides with <80% purity were purified by automatic flash chromatography using SNAP 12 g KP-C18-HS cartridges in an ISOLERA ONE (BIOTAGE). A gradient of CH_3_CN:H_2_O (0.05% TFA) from 0:100 to 30:70 over 60 min as mobile phase, and a flux of 5 mL/min were used. Peptide purity was analyzed using an analytical HPLC: Waters (model 2690) with a SUNFIRE^TM^ column C18 (3.5 m, 4.6 × 50 mm) at 1 mL/min with a 5 to 50% gradient of CH_3_CN (0.08% HCO_2_H):H_2_O (0.01% HCO_2_H) in 15 min as mobile phase or Agilent (model 1120 Compact LC) with Eclipse Plus column C18 (4.6 × 150 mm) at 1.5 mL/min with a 5 to 50: gradient of CH_3_CN:H_2_O (0.05% TFA) in 20 min as mobile phase. Characterization of the products was performed by HPLC-MS (Waters, Milford, MA, USA) coupled to a single quadrupole ESI-MS (Micromass ZQ 2000, Waters, Milford, MA, USA.

Detailed experimental procedures for the conformational analysis of the peptides (CD and NMR) to corroborate their tendency to adopt the desired helical structure are gathered in the Supplementary Material of [15].

### 3.2. Chemiluminescent Competition Assays

As described by Muller Y. A. et al. [19], the surface of white high-binding 96-well microplate (Corning Life Sciences, Amsterdam, The Netherlands) was coated with 100 μL of phosphate-buffered saline solution (PBS, pH 7.4) containing 200 ng/mL of VEGFR-1 D1-D7 (ECD domain)/Fc Chimera or 150 ng/mL of VEGFR1 D1-D3/Fc chimera (Bio-techne R&D, Abingdon, UK) and incubated at 4 °C overnight. After three washes with 250 μL of PBS 0.1%, (*v*/*v*) tween 20 (buffer A), the plate was blocked by 200 μL of PBS with 3% (*w*/*v*) of BSA and stirred at 37 °C for 2 h. The plate was washed three times with buffer A. Then, 100 μL of a solution containing 131 pM of btVEGF_165_ (Bio-techne, R&D, Abingdon, UK) and the tested compounds at various concentration diluted in PBS containing 1% DMSO were added in each well. After 3 h stirring at 37 °C, the plate was washed four times with buffer A and 100 μL of streptavidin-horseradish peroxidase (Amersham Biosciences, Little Chalfont, UK) diluted at 1:8000 in PBS containing 0.1% (*v*/*v*) Tween 20 and 0.3% (*w*/*v*) BSA were added per well. After 1 h of incubation at 37 °C under obscurity and stirring, the plate was washed five times with 250 μL of buffer A and 100 μL of SuperSignal West Pico chemiluminescent substrate (Pierce, Appleton, WI, USA) were added. The remaining bt-VEGF_165_ was detected by chemiluminescence, which was quantified. The percentages of btVEGF_165_ displacement were calculated by the following formula: percentage of displacement = 100 × [1 − (S − NS)/(MS − NS)], where S is the signal measured, NS is the nonspecific binding signal and MS is the maximum binding signal observed with btVEGF_165_ without compounds tested. Either peptides were tested at 100 μM to determine a displacement percentage, or in a dose–effect relationship to determine their IC_50_ using the nonlinear regression function in Prism (GraphPad software, La Jolla, CA, USA). Each experiment was performed three times in triplicate.

## Figures and Tables

**Table 1:** Inhibitory potency of the previously described peptides Ac-SSEE**X^5^**ARN**X^9^**AA**X^12^**N-NH_2_ on VEGFR1 ^a^.

Compd. No.	Compd. ^b^ X^5^X^9^X^12^	% of Displacement ^c^ (100 μM)	IC_50_ (µM) ^d^
**1**	FYY	29 ± 3	ND ^f^
**2**	FYW	48 ± 6	29 ± 5
**3**	FWY	50 ± 5	23 ± 4
**4**	WYY	45 ± 4	>100
**5**	FFY	39 ± 6	>100
**6**	YYY	33 ± 4	ND
**7**	FYI	n.a. ^e^	ND
**QK ^g^**	-	69 ± 3	32 ± 8 ^g^

^a^ Displacement assays; ^b^ Compounds already described and tested in our previous study; ^c^ Activity corresponds to the percentage of biotinylated VEGF_165_ displaced by a 100 μM concentration of peptide on the whole extracellular domain (ECD, D1–D7) of VEGFR1; ^d^ Relative inhibitory concentration 50; ^e^ n.a refers to no significant activity; ^f^ ND = Not determined; ^g^ Described VEGFR1 binder peptide, Ac-KLTWQELYQLKYKGI-NH_2_ [21] (value from [15]).

**Table 2:** Inhibitory potency of selected peptides Ac-SSEE**X^5^**ARN**X^9^**AA**X^12^**N-NH_2_ on VEGFR1 ^a^.

Compd. No.	Compd. X^5^X^9^X^12^	% of Displacement ^b^		
		100 μM	30 μM	30 μM + EDTA
**8**	YWW	63 ± 2	63 ± 1	40 ± 7
**9**	FFF	37 ± 3	47 ± 2	- ^d^
**10**	WFF	55 ± 1	64 ± 3	55 ± 4
**11**	YFF	7 ± 4	5 ± 3	-
**12**	FWF	55 ± 1	66 ± 1	53 ± 4
**13**	WWF	58 ± 5	50 ± 4	34 ± 3
**14**	YWF	58 ± 1	62 ± 4	56 ± 6
**15**	FYF	27 ± 5	5 ± 4	-
**16**	WYF	62 ± 1	71 ± 2	44 ± 5
**17**	YYF	6 ± 1	n.a ^c^	-
**18**	WFW	65 ± 2	61 ± 1	31 ± 2
**19**	YFW	54 ± 3	37 ± 1	-
**20**	FWW	43 ± 3	41 ± 3	-
**21**	WWW	78 ± 2	62 ± 6	21 ± 4
**22**	FFW	82 ± 1	63 ± 2	19 ± 3
**23**	WYW	50 ± 1	28 ± 2	-
**24**	YYW	70 ± 2	41 ± 2	n.a
**25**	WFY	43 ± 5	22 ± 3	-
**26**	YFY	38 ± 4	12 ± 5	-
**27**	WWY	64 ± 5	57 ± 3	32 ± 4
**28**	YWY	44 ± 4	44 ± 2	-
**29**	FWI	72 ± 1	60 ± 3	71 ± 2
**30**	FIW	47 ± 6	29 ± 7	-
**31**	FYL	n.a	n.a	-
**32**	FIY	60 ± 1	59 ± 4	55 ± 1
**33**	FLY	19 ± 7	6 ± 4	-

^a^ Displacement assays; ^b^ Activity corresponds to the percentage of biotinylated VEGF-A displaced by a 100 or 30 μM concentration of peptide on the whole extracellular domain (ECD, D1–D7) of VEGFR1; ^c^ n.a refers to no significant activity; ^d^ means that the measure was not performed [15].

**Table 3:** IC_50_ values of selected compounds. Displacement assays.

Entry	Compd. X^5^X^9^X^12^	IC_50_^a^ (μM) (95% Confident Interval)
**8**	YWW	25.6 [18.6–35.1]
**10**	WFF	14.5 [9.4–22.2]
**12**	FWF	48.6 [31.8–74.3]
**14**	YWF	14.0 [10.0–19.6]
**16**	WYF	21.3 [13.9–32.7]
**29**	FWI	4.6 × 10^−2^ [1.4 × 10^−3^–1.5]
**32**	FIY	30.4 [19.4–47.5]

^a^ Relative inhibitory concentration 50.
